# Tactical-Grade Wearables and Authentication Biometrics

**DOI:** 10.3390/s26030759

**Published:** 2026-01-23

**Authors:** Fotios Agiomavritis, Irene Karanasiou

**Affiliations:** Department of Military Science, Sector of Mathematics and Engineering Sciences, Hellenic Army Academy, 16672 Vári, Greece; fagiomavritis@tuc.gr

**Keywords:** wearables, biometric authentication, operational resilience sensors, electrocardiography, electroencephalography, body sensor networks, internet of battlefield things

## Abstract

Modern battlefield operations require wearable technologies to operate reliably under harsh physical, environmental, and security conditions. This review looks at today and tomorrow’s potential for ready field-grade wearables embedded with biometric authentication systems. It details physiological, kinematic, and multimodal sensor platforms built to withstand rugged, high-stress environments, and reviews biometric modalities like ECG, PPG, EEG, gait, and voice for continuous or on-demand identity confirmation. Accuracy, latency, energy efficiency, and tolerance to motion artifacts, environmental extremes, and physiological variability are critical performance drivers. Security threats, such as spoofing and data tapping, and techniques for template protection, liveness assurance, and protected on-device processing also come under review. Emerging trends in low-power edge AI, multimodal integration, adaptive learning from field experience, and privacy-preserving analytics in terms of defense readiness, and ongoing challenges, such as gear interoperability, long-term stability of templates, and common stress-testing protocols, are assessed. In conclusion, an R&D plan to lead the development of rugged, trustworthy, and operationally validated wearable authentication systems for the current and future militaries is proposed.

## 1. Introduction

Integration of wearable sensing technology in military applications has revolutionized the capture of physiological, behavioral, and environmental information in real time. In comparison to commercial-level wearables, military-level wearables are designed intentionally to function under extreme environments, including high motion, thermal stresses, electromagnetic perturbation, and physical shock, with ensured secure and reliable operation. Notably, the underlying sensing, signal-processing, and materials innovations exhibit significant dual-use applicability, supporting translational deployment in civilian sectors such as disaster response, hazardous-industry monitoring, and performance optimization. Moreover, the systems are being integrated increasingly with biometric verification techniques to provide verification of identity, health control, and security improvement of operations [1].

Biometric verification is an appealing substitute for traditional credentials in battlefield scenarios, in which passwords, tokens, or cards are not practical or vulnerable. Biological and behavioral measures such as electrocardiogram (ECG), photoplethysmogram (PPG), and electroencephalogram (EEG), and behavioral biometrics such as gait and voice may be passively acquired and utilized for identity verification [2,3]. Implemented within wearable systems, modalities give unobtrusive, on-body verification without disruption of mission flow.

However, the use of biometric systems in extreme condition settings presents unique challenges. Physiological variation caused by fatigue, dehydration, or injury can compromise recognition accuracy [2]. Harsh environmental factors and equipment interferences can create noise or disable sensor operation [1]. In addition, security threats such as spoofing, replay attacks, and template capture require the establishment of formidable countermeasures, especially when biometric information is analyzed locally on hardware [4]. Directives such as NIST SP 800-63-4 [5] and ISO/IEC 30107 [6] provide templates for secure integration and testing.

In this review, tactical-grade wearable technologies and biometric authentication systems are examined with respect to sensing innovations, operational limitations in real-world conditions, security paradigms, and future research directions. While biometric modalities have been extensively investigated in isolation, there remains a notable lack of comprehensive reviews that assess their performance and reliability under stressful, adversarial, and tactically complex operational conditions. This gap has become increasingly important as contemporary military environments deploy wearable technologies that were often not originally designed for sustained operation under extreme physiological stress, environmental exposure, and security threats, thereby leading to degraded performance and increased security risk.

This work aims to address this gap by synthesizing wearable sensing technologies and biometric authentication modalities within a unified operational military framework. By identifying limitations, risks, and deficits in operational robustness and security, the review seeks to inform future research and development efforts toward the design of accurate, dependable, and secure biometric verification systems suitable for realistic battlefield deployment.

Within the comparative analysis presented throughout this manuscript, the summary tables focus on modality-specific characteristics that are most relevant to biometric authentication performance. Beyond these core factors, overall system effectiveness is also influenced by environmental conditions, sensor placement, integration constraints, and operational usability. As these aspects vary substantially across biometric modalities and deployment scenarios, they are addressed qualitatively in the relevant sections of the text rather than through direct tabular comparison.

### 1.1. Review Methodology

For the purposes of carrying out a structured and thorough analysis, the literature was searched using IEEE Xplore, PubMed, Scopus, and Google Scholar, complemented by standards repositories (ISO/IEC and NIST) and NATO technical reports. Search terms included combinations of “biometric authentication,” “wearable sensors,” “tactical grade sensors,” “ECG,” “EEG,” “PPG,” “IMU,” and “presentation attack detection.” The literature searched was primarily published between 2014 and 2025. The inclusion criteria focused on peer-reviewed journal articles, international standards, and authoritative technical reports that addressed biometric authentication or wearable sensing under realistic operational, high-stress, or field-deployed conditions. Studies limited to controlled laboratory environments without consideration of motion artifacts, environmental stressors, or deployment constraints were excluded, unless they introduced foundational methods directly applicable to tactical contexts. The selected works were qualitatively analyzed and grouped according to biometric modality, sensing technology, operational challenges, and security considerations. This approach enabled the identification of dominant trends, persistent limitations, and research gaps relevant to military-grade biometric authentication systems.

### 1.2. Manuscript Structure

The manuscript is organized to distinguish between wearable sensing technologies and biometric authentication methodologies in tactical environments. Section 2 reviews tactical-grade wearable sensor platforms, focusing on physiological, kinematic, and environmental sensing technologies and the operational constraints affecting their deployment. Section 3 builds upon this foundation by examining biometric authentication modalities derived from these sensor systems, assessing their suitability, robustness, and limitations in military contexts. Section 4 discusses operational challenges arising from physiological variability, environmental stressors, motion artifacts, and equipment interference. Section 5 addresses security, privacy, and governance considerations, including template protection, spoofing resistance, adversarial threats, and ethical implications. Finally, Section 6 outlines future research directions and strategic pathways, emphasizing multimodal integration, edge computing, and standardization needs.

### 1.3. Definition of Tactical-Grade Wearables

For tactical-grade applications, biometric and sensing technologies must operate reliably under operational conditions that include motion artifacts and limited communication resources. Unlike commercial wearables, which emphasize comfort and usability, tactical-grade solutions must prioritize security and dependable performance. Key challenges affecting biometric authentication in tactical environments include performance limitations, resource constraints, interoperability, and trust and security requirements. These issues are discussed throughout this review.

## 2. Tactical-Grade Wearable Technologies

Tactical-grade wearables should not be regarded as commercial devices that have simply been reinforced for durability. They are engineered from the outset to maintain measurement accuracy and operational reliability under demanding conditions, including wide temperature variations, electromagnetic interference, and high-gravity maneuvers. In the sections below, we provide an overview of the major sensor categories, their operational duties, and the challenges involved in their integration within defense applications.

The analysis presented in this review is theoretical and is derived from the experimental results reported in the existing literature. No new experiments were conducted. The comparative tables presented herein emphasize modality-specific strengths and limitations relevant to authentication performance, starting with Table 1 that summarizes the main categories of sensors used in military wearables which the subsequent subsections expand on. However, additional system-level variables, including power efficiency, computational complexity, communication latency, and integration effort, strongly influence the feasibility of multimodal biometric systems in tactical environments. These aspects are addressed qualitatively across Section 2 and Section 4, as their quantitative comparison is highly dependent on platform architecture and mission context.

### 2.1. Physiological, Neurophysiological Signals and Wearable Sensing Systems

**Figure 1:**
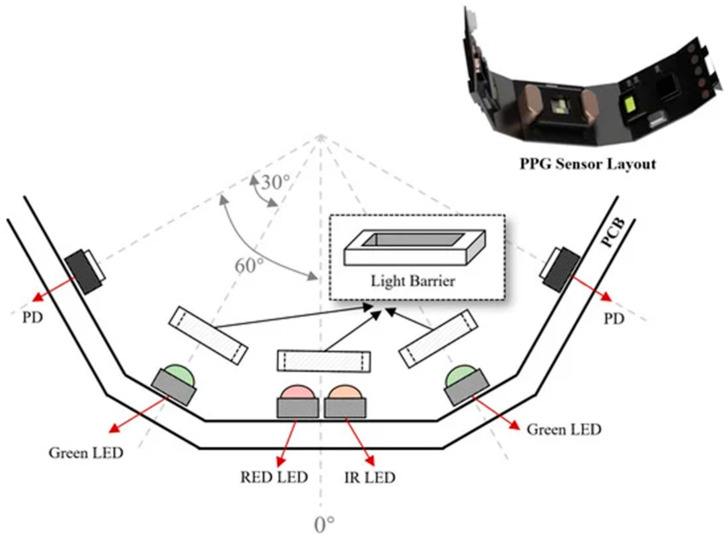
Proposed PPG sensor layout illustrating placement and signal acquisition for wearable photoplethysmography. Reprinted from Ref. [9], distributed under the terms of the Creative Commons Attribution License (CC BY 4.0).

**Figure 2:**
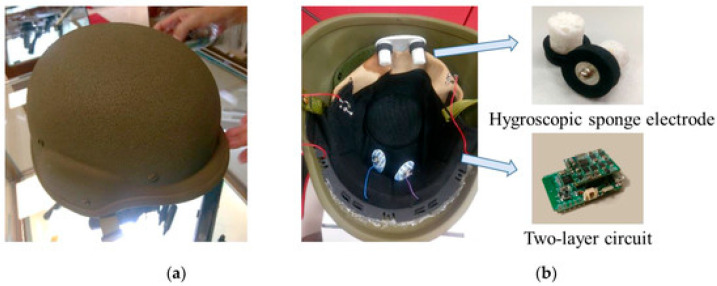
Wearable EEG-based smart helmet designed for strategic brain–computer interface applications, showing integrated sponge electrodes and embedded signal-processing circuitry. (**a**) Top view (**b**) Interior view. Reprinted from Ref. [10], distributed under the terms of the Creative Commons Attribution License (CC BY 4.0).

Physiological and neurophysiological signals, acquired via wearable sensing modalities, support soldier health monitoring and biometric identification systems by measuring internal biological signs indicative of identity and operational states [7,8]. Their use in real-world situations requires robustness to motion artifacts, environmental noise, and physiological variability [9,10]. The main categories are as follows:

- Electrocardiogram (ECG) acquisition sensors: ECG is also well researched as both a health monitor and a biometric modality. Its morphological characteristics (QRS complex, P-wave, and T-wave) are very individual and allow for continuous verification. Nevertheless, movement artifacts and electrode position variability continue to pose significant issues for use in battlefield situations. Current reviews stress AI-driven denoising and adaptive filtering for enhancing robustness [7].
- Photoplethysmography (PPG) optical sensors: PPG sensors, in many cases included in wristbands, rings (as illustrated in Figure 1), or chest straps, estimate blood volume variation. They are lightweight and energy-efficient but extremely sensitive to movement and variability in perfusion. Development using multi-wavelength PPG and integration using accelerometer data has enhanced the accuracy during exertion [8,9].
- Electroencephalography (EEG) acquisition sensors: EEG provides indicators of cognitive workload, fatigue, and stress. Current work includes the integration of dry electrodes into helmet systems for real-time monitoring of neural activity [9,10]. However, EEG signals are highly susceptible to motion-induced artifacts, requiring careful mechanical and electrical design of the headgear to minimize noise contamination. Figure 2 provides an example of an EEG-based acquisition sensor, a smart helmet designed for strategic brain–computer interface applications.
- Electromyography (EMG) acquisition sensors: Muscle activation is measured by EMG acquisition sensors for use in exoskeleton control and fatigue monitoring. Field tests indicate that EMG may predict musculoskeletal strain, but reliability decreases due to sweat, electrode movement, and armor interference [11].

Existing precision health systems employ physiological and neurophysiological sensors for the early detection of heat injuries, dehydration, and fatigue. However, continuous monitoring of service members’ health states also introduces significant privacy and ethical considerations [12].

### 2.2. Kinematic Sensors

Kinematic sensors have the vital role of monitoring movement, posture, and behavior in real time. Inertial measurement units (IMUs) consist of accelerometers, gyroscopes, and magnetometers. IMUs are indispensable for gait biometrics, GPS-denied navigation, and activity recognition. Contemporary MEMS-based IMUs are small but exhibit drift and susceptibility to magnetic disturbances. Current sensor fusion algorithms incorporate IMU data along with GPS, vision, or PPG in order to provide more accuracy [3,12].

In tactical environments, inertial measurement units (IMUs) can be classified as consumer-grade, industrial-grade, and tactical-grade IMUs based on bias stability, noise, and long-term drift behavior. Since tactical-grade IMUs use more precise inertial sensing to counter long-term drift, which would be highly desirable in applications that use human gait or motion as a means to carry out biometrics, this capability would be critical in a military setting. Various methods that help counter drift, such as Kalman filtering, complementary filtering, zero-velocity update, and multi-sensor fusion (using magnetometers and GNSS, if available), would be used to help cope with dynamics on a tactical field environment [3,12]. These mitigation strategies are central to enabling reliable IMU-based gait biometrics and navigation in high-dynamics tactical environments.

In military applications IMUs support several mission-critical functions, such as the following:

- Navigation: IMUs allow the soldiers access to their equipment in GPS-denied environments, an expanding threat for electronic warfare [12].
- Authentication: Gait signatures extracted from the IMUs are also being experimented with as continuous biometric signatures [3].
- Exoskeletons: Stability control and load distribution receive inputs from the IMUs.

IMUs are invaluable for situational awareness, but long-term drift and vibration sensitivity continue as challenging issues for battlefield use.

### 2.3. Environmental Sensors

Environmental monitoring plays an important role in situational awareness and combatant safety [13]. One of the key aspects of environmental monitoring is tracking heat stress/dehydration risk, which is measured by temperature/humidity indicators collected by environmental sensors. Other aspects of environmental monitoring include gas sensor networks (CO, NO_2_, SO_2_, and H_2_S) integrated into uniforms for measuring toxic exposure risk [13]. The other key aspect would be CBRN(Chemical, Biological, Radiological, and Nuclear) protective suits that use radiation sensors for tracking dangerous levels of exposure.

Recent advances in nano-based gas sensors enable lightweight, flexible, and highly selective battlefield pollutant detection [14]. Next-gen uniforms now integrate IoT-enabled sensor arrays for real-time environmental and physiology monitoring [15]. Wearables beyond health are extended by environmental sensors into threat detection, but pose challenges of sensor calibration drift and power consumption due to continuous monitoring.

### 2.4. Multimodal Platforms

Contemporary defense research places strong emphasis on sensor fusion, integrating physiological, kinematic, and environmental data into a coherent operational picture [1,7,16]. Representative developments include Body Sensor Networks (BSNs), which are distributed sensor networks on the battlefield on the soldier’s body that allow for redundancy and robustness [1,7]. Another development is edge computing, which can be defined as an Artificial intelligence-driven merge at the tactical edge that reduces latency and preserves functionality in communications-denied situations [16].

**Figure 3:**
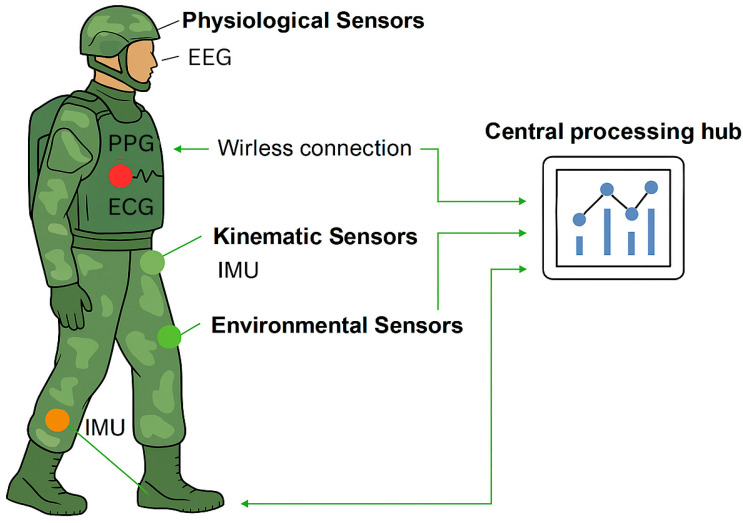
Conceptual layout of military wearable sensor network.

These technology trends parallel the broader drive for military transformation with a focus on sensing-integration and decision distribution. Certain programs in the U.S. Army, for example, such as the “Future Soldier” vision, involve a “soldier-centric” framework for the real-time fusion of physiological, kinematic, and environmental information to support preparedness, survivability, and effectiveness. At the operational level, the NATO CJADC2 concept promotes the integration of sensing, communication, and command and control operations across all domains, requiring wearable systems to be resilient IoBT nodes with the capability to provide real-time multimodal inputs to joint situational awareness [16]. In concert, these programs reflect the operational imperative for multimodal platforms, confirming the need for standardized architectures and effective data fusion pipelines in future tactical wearable systems addressing the broadened spectrum of tactical operations under conditions of future conflict scenarios [1,7,16]. Future tactical wearables will incorporate multimodal sensing platforms, but their effectiveness will depend on interoperability across equipment types, military services, and coalition partners. Achieving this will require the development of standardized architectures and secure data-fusion protocols. Building on the architectural principles discussed above, Figure 3 shows the conceptual layout of the military wearable sensor network and its main functional components

## 3. Biometric Modalities for Authentication

For military applications, biometric authentication requires modalities showing resistance to duress, imperviousness to spoofing, and suitability for continuous use. Unlike civilian settings, the battlefield imposes distinct operational constraints: personnel must carry substantial loads, function under extreme environmental conditions, and must maintain security without hindering operational effect. The following subsections compare the most relevant biometric modalities for defense applications with respect to their strengths, limitations, and relevance in operational applications. To support this analysis, Table 2 summarizes the key biometric modalities considered in this section.

### 3.1. Cardiac Biometrics (ECG and PPG)

Cardiac signals have become potential biometric identifiers because of their inherent uniqueness and the capability for real-time monitoring. Signals derived from the electrical activity of the heart in the form of electrocardiogram (ECG) signals present highly distinctive morphological patterns that can be used for authentication. Under laboratory conditions, deep learning classifiers have attained symmetric error rates below 2%, thus proving the viability of ECG as a reliable biometric [17,18]. Application in battlefield scenarios, however, requires dealing with motion artifacts, variability in the position of electrodes, and physiological changes caused by the effect of stress, all of which can warp the signal.

Photoplethysmography (PPG), which measures changes in blood volume through the means of optical sensing, is the less invasive alternative. When deployed in wristbands or gloves, PPG provides the capability for low-power, real-time monitoring; it is, nonetheless, highly susceptible to motion noise and perfusion variations [18,19]. Recent developments in multi-wavelength sensing and sensor fusion with accelerometers enabled the enhancement in robustness; both ECG and PPG, however, remain vulnerable to the physical strains of battlefield activities.

### 3.2. Neurophysiological Biometrics (EEG)

EEG offers an entirely different method of authentication with the recording of brainwave activity. Patterns detected in the alpha, beta, and gamma EEG frequency bands can serve as biometric signatures, offering strong resistance to impersonation and robustness against surface-level spoofing attacks. This makes the EEG interesting for applications with high security where resistance to adversary attempts is at a premium [20]. There are, however, significant obstacles that currently limit practical implementation in operational settings. Integration in helmets is highly technical, signal quality is readily compromised by movement and environmental interference, and the research in ethics of observing cognitive states is compromising autonomy and privacy. Although EEG-based biometrics represent a promising area of research, their practical implementation in authentication systems, particularly in operational military settings, remains limited.

Nevertheless, improvements in dry electrode materials, artifact-reduction algorithms, low-power signal processing, and compact head-mounted systems are gradually reducing the barriers posed by motion noise, environmental stressors, and user burden [20]. In parallel, progress in adaptive modeling and multimodal fusion may allow EEG features to complement other physiological or kinematic signals, improving robustness without requiring full-scale neural monitoring [21]. As these developments converge, EEG could transition from a predominantly experimental modality to a viable component within future tactical authentication architectures.

### 3.3. Muscular Biometrics (EMG)

Electromyography (EMG) records muscle activation patterns that are characteristic of individuals and lend themselves to authentication. In military applications, EMG is of specific value for exoskeleton control, in which movement intentions can be predicted from muscle signals, and for detecting fatigue, in which reduced muscle activity may signal decreased performance [19]. As a biometric modality, EMG benefits from its direct correspondence to neuromuscular activation patterns, making it difficult to replicate artificially. However, its practical reliability is compromised by factors such as sweat-induced signal degradation, electrode displacement, and interference from protective clothing, which collectively limit its viability for consistent operational use. The restrictions at present limit the role of EMG to supplementary rather than prime authenticators, though future developments in dry electrodes and clothing-based sensors can be expected to widen the scope.

### 3.4. Behavioral Biometrics (Gait and Voice)

Behavioral features like gait and speech patterns constitute covert techniques of authentication that may be used continuously without explicit user input. Gait analysis, commonly with data provided by inertial measurement units (IMUs), has proved significantly promising for military applications, particularly in the task of navigation in GPS-denied areas and for continuous identification [3,22]. Loads carried, uneven terrain, and body fatigue may negatively impact the efficacy of gait signatures in deployed scenarios.

Voice verification offers another natural interface for hands-free submission in scenarios where manual input is inconvenient. Nevertheless, the integrity of speech signals can be compromised by tampering and by background noise levels characteristic of operational environments [21,23]. All the same, behavioral biometrics remain interesting because of the covertness of the features and the possibilities for embedding them seamlessly in existing communication platforms.

### 3.5. Multimodal Fusion

Due to the shortcomings of single modalities, multimodal integration has become the most promising method of military authentication [24]. Through the integration of signals like ECG, gait, and voice, for instance, the strengths of each modality can be compensated for and more trustworthy authentication provided [1,22]. At the feature, score, or decision level, the fusion can be performed with AI-based methods increasingly chosen to improve performance. In battlefield applications, multimodal systems also ensure continuity of proof-of-life even in the event of modality compromise—for example, the interference of PPG sensors by gloved hands or the interference of the human voice by background noise. This modality redundancy is essential in providing both operational efficacy and security in the face of risky operating conditions.

## 4. Operational Challenges in Defense Contexts

**Figure 4:**
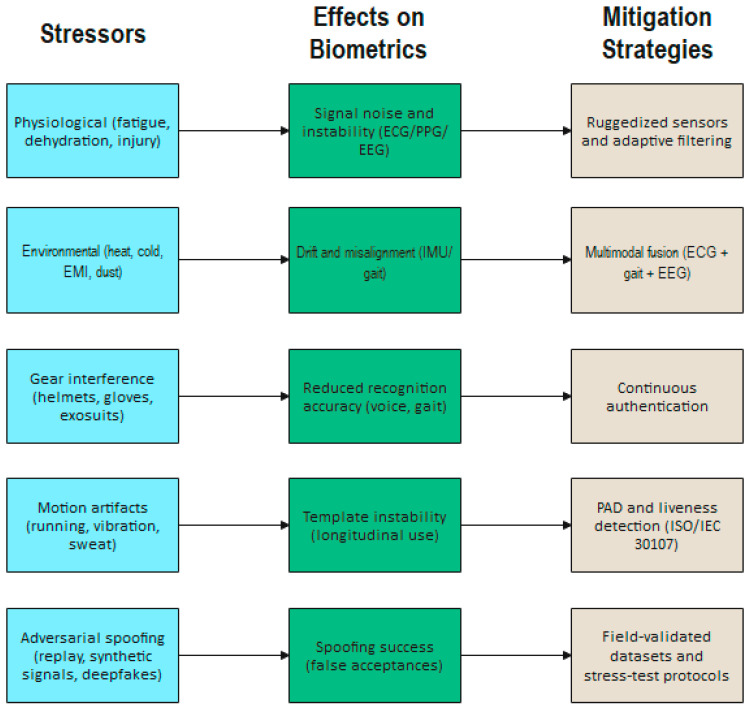
Biometric system vulnerabilities and mitigation pathways in military contexts.

Biometric authentication systems face a unique set of challenges when introduced into a defense environment. Accuracy can be achieved with lab experiments; however, working under different environmental conditions affects accuracy and reliability. This section aims to compile existing knowledge and recent developments that highlight defense-related challenges to achieving reliable field-ready biometric systems. A system-level view of these vulnerabilities and their associated mitigation pathways is provided in Figure 4.

### 4.1. Physiological and Behavioral Variability

Biometric characteristics vary with the operator’s physical and mental state. Conditions of fatigue, dehydration, or physical injuries result in changes to ECG patterns, EEG power spectra, and gait cycles [7,17,18]. Fatigue affects EEG alpha power and reaction times by making it difficult to set a proper authentication level [7]. Gait patterns are affected by loading and musculoskeletal pain due to reduced accuracy of recognition during actual battlefield scenarios [3,22]. Voice recognition can easily be affected by stress-related changes in pitch and tone, which can often be found during a battlefield environment [23]. Current reviews highlight that a continuous authentication system should be able to counter such changes to some effect [25].

### 4.2. Environmental Extremes

Military deployments test sensors against heat, cold, dust, moisture, and electromagnetic interference (EMI). Heat and dryness degrade peripheral perfusion, making PPG a weaker signal source [18,19]. Cold hardens tissue, which impacts EMG patterns and gait patterns as well [3]. Dust and moisture can disrupt sensor-to-skin contact; EMI generated by vehicles and communications gear can saturate EEG/ECG signals [20]. Also indicative of the challenge of environmental adaptation for sensors is a 2023 IEEE survey of the need for algorithms to tolerate environmental interference [25]. Indeed, multimodal fusion has been suggested to counter modality failures when exposed to environmental stress factors [24,26].

### 4.3. Gear Interference

Standard military equipment often interferes with the ability to capture biometrics. For example, helmets and visors can interfere with collecting the EEG electrodes and voice sensors, gloves and exosuits can interfere with PPG and EMG [8,12], and load-bearing vests displace chest strap ECG sensors from their proper location [11]. In addition to load-bearing vests, there is also the possibility of smart uniforms, which may hold promise for integrated monitoring but can introduce motion artifacts and alignment issues if not properly considered in their design [12,14]. Recent research on gait recognition based on the analysis of footstep data suggested that unobtrusive authentication was possible with the footstep restrictions of military equipment [26]. To support the assessment of modality robustness in real operational settings, Table 3 lists common forms of gear interference and their effects on biometric signal quality.

### 4.4. Motion and Activity Artifacts

High-intensity activities (running, crawling, and firing) introduce motion artifacts across modalities. PPG is especially sensitive to motion noise, while EEG electrodes lose contact under vibration [20]. IMU-based gait recognition is already affected by drift under dynamic load carriage [3,22]. Sweat accumulation is an additional factor that affects electrode-skin impedance [7]. A 2024 multimodal study reports that besides the single-modality failures, fusion approaches are the most efficient way to deal with these problems [27].

### 4.5. Field Validation and Dataset Gaps

The majority of biometric systems are tested on laboratory-grade data sets that do not incorporate conditions seen on the battlefield [2,17]. There are no accepted methods of stress-testing or field-validated data sets that target variations in fatigue, environmental extremes, and equipment interference [7]. The endurance of the biometric template over time is a quest not taken yet [18,22]. New explorations pointed out the demand for authentic longitudinal data sets to guarantee operational functionality [28]. Since the market has no longitudinal data sets to offer, research and claims concerning toughness become speculative.

### 4.6. Spoofing and Adversarial Manipulation of Sensors

Spoofing attacks are an emerging challenge for biometric applications working in a hostile environment. Unlike environmental noise, spoofing requires planned manipulation of bio-inputs to imitate or cheat an authentication procedure [4]. In a military setting, attackers may attempt to misuse vulnerabilities found in ECG sensors, PPG sensors, or gait sensors by broadcasting artificial bio-inputs to imitate physiological patterns to interrupt authentication procedures by emitters that attackers may wear [19,25]. ECG/PPG spoofing studies have shown that attackers can easily defeat unimodal ECG/PPG authentication systems by replaying or simulating waveform patterns [16,17]. In a reality environment, attackers may take advantage of wireless communication channels or sensor contact integrity to inject spoofing patterns. Furthermore, in Gait spoofing, the gait recognition system is susceptible to adversarial imitation or exoskeleton-powered robots that imitate a soldier’s gait pattern [22,26]. Another major threat is voice spoofing, in which the attacker utilizes voice replay/deepfake technology as a means of attacking existing voice biometric authentication procedures, and it has been documented enough in civilian applications alone [23]. In multimodal systems, while multimodal fusion reduces the risk of single-channel spoofing, it also increases the attack surface, requiring robust anti-spoofing protocols [27].

Recently, researchers have stressed the importance of presentation attack detection (PAD) and liveness detection as part of the operation environment itself [6,25,28]. For instance, deep learning algorithms for PAD with the ability to spot small anomalies of spoofing signals have been suggested for design purposes only [29]. In reality, however, adaptation to edge AI operation for wearables needs to be accomplished by those algorithms.

### 4.7. Summary

Challenges arising from operation scenarios indicate the vulnerability of biometric authentication systems to both incidental stress sources and adversarial threats. Factors of physiological variations (fatigue, dehydration, and injuries), environmental conditions (heat, cold, EMI, and dust), and system interference (helmet, gloves, and exosuit) weaken the received signal and system efficiency [3,7,8,13,16,17,18,19,20,22,23]. Person motion and sweating further disrupt sensor touching points, while untested datasets for operation scenarios weaken system integrity [2,7,17,18,22,28].

Apart from these natural sources of stress, spoofing and adversarial attacks can be viewed as a specific operational challenge. Replay attacking, signal injection, gait spoofing, and voice imitation attacks can target unimodal systems and hence require presentation attack detection (PAD) and liveness detection in their defense setup [4,6,23,25,27,29].

Overall, these requirements highlight the need for hardened sensors and algorithms that can support multimodal fusion for validation. This discussion leads into Section 5, which addresses spoofing and adversarial attacks related to privacy and security.

## 5. Security and Privacy in Military Biometric Authentication

The challenges identified in Section 4 serve to highlight just how susceptible biometric systems can be to stress. But aside from the challenges entailed in a humid, variable, or extreme environment, and those presented by human physiology, another set of challenges can threaten the implementation of biometric authentication within the military. These include template security, spoofing, adversarial machine learning, and ethical issues. To contextualize these challenges, Table 4 outlines the principal security and privacy threats in military biometric authentication together with the countermeasures designed to address them.

### 5.1. Template Security and Storage Risks

Biometric templates, once rendered insecure, can never be revoked, as passwords can be. In the military context, template theft or reversal could potentially be used for spoofing or creating vital physiological information [17,18]. Centralized systems, as efficient as they may be, form a point of attack susceptible to cyber threats [28]. A distributed approach could avoid such points of attack but would be plagued by synchronization issues [30]. The use of cancelable biometrics or homomorphic cryptography has recently gained popularity as a means of making template manipulation or processing encrypted information without resorting to raw data [30]. Unfortunately, such operations are computationally intensive, making it difficult to implement a portable, low-power system.

### 5.2. Spoofing Countermeasures and PAD Integration

As explained in Section 4.6, spoofing attacks use sensor vulnerabilities based on replay attacks of ECG/PPG recordings, gait spoofing, or voice deepfakes [4,23,26]. To mitigate these attacks, presentation attack detection (PAD) has emerged as a vital component of biometric security, formalized in the ISO/IEC 30107 standards [6]. PAD can be implemented using liveness detection, which can be conducted based on pulse oximetry for PPG, micro-movement assessment for gait, challenge-response tests for voice biometrics, and so on [25]. Deep learning-based PAD systems have demonstrated potential in identifying small discrepancies in spoofed attacks [29]. In military scenarios, PAD should be light, robust, and adaptable to multimodal fusion biometric systems.

### 5.3. Adversarial Machine Learning Threats

The use of deep neural networks in biometric characterization has become susceptible to attacks from adversarial machine learning (AML). Well-designed attacks can be used to misclassify biometric data, thereby facilitating unauthorized entry [31]. Inversion attacks can be used to reverse-engineer biometric characteristics from the neural networks trained, while Data Poisoning attacks can result in reduced performance over time [31]. For a military environment, attacks could be specifically designed to generate Adversarial Signals to overcome authentication and create denial of service scenarios. Adversarial Robust Training, Defensive Distillation, and Input Sanitizing have been researched, though often at the expense of accuracy [31].

### 5.4. Privacy and Ethical Considerations

The question remains as to whether continuous biometric observation can be considered acceptable in relation to privacy, freedom, and consent. Biometric systems designed to detect fatigue, stress, or cognitive loads could potentially be used for spying on the mental conditions of troops [7,23]. Even though biometric observation can be very useful in improving the security of troops, it can potentially be used as a means of personnel control, which could violate human rights [32]. Balancing defense security along with human rights remains a challenge.

### 5.5. Standards, Governance, and Interoperability

International standards organizations, such as ISO/IEC JTC 1/SC 37, have set guidelines on the protection of biometric data, template security, and PAD [6,33]. The NATO community has also specified directives on secure identity management, although military-specific norms on biometric authentication are scattered. The lack of norms stress-tested within a real-world context has made it difficult for interoperability and trust among forces. The development of norms on secure deployment, spoof resistance, and ethical regulation has become vital [33].

### 5.6. Summary

Security and privacy challenges go beyond the operational issues discussed in Section 4. In military applications, biometric systems face risks such as template theft, spoofing attacks, adversarial machine learning, and overly intrusive surveillance. These challenges can be addressed through a multi-tiered approach:

- Technical measures such as cancelable biometrics, encrypted matching, and presentation attack detection (PAD).
- Improving the robustness of biometric algorithms against adversarial manipulation.
- Ethical governance to support fair and transparent use.
- Harmonization of practices across defense organizations through standardization.

**Figure 5:**
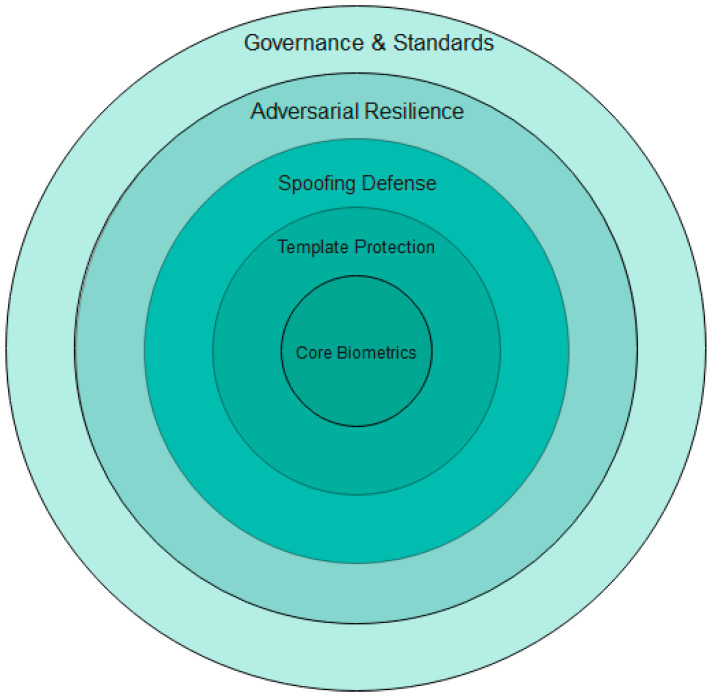
Security and privacy layers in military biometric authentication.

Together, these elements are necessary for implementing biometric authentication in a secure, ethical, and effective manner within contested environments. To consolidate these considerations, Figure 5 illustrates the layered security and privacy framework needed for secure authentication in operational settings. This discussion leads into Section 6, which focuses on future directions for military biometric systems.

## 6. Future Directions and Strategic Pathways in Military Biometric and Wearable Technologies

The preceding sections examined key technical obstacles, security risks, and governance considerations associated with military-grade biometric systems. Given the pace of current technological development, a perspective focused on future developments is required. This section, therefore, focuses on emerging technologies, military-grade wearable systems, and strategic considerations related to system design, ethics, and future research directions within the broader defense technology landscape.

### 6.1. Emerging Modalities and Biometric Innovations

Recent breakthroughs in multimodal fusion have shown the effectiveness of integrating physiological features (ECG, EEG, PPG) with behavior patterns (gait, voice, keystroke patterns) to improve stress robustness [34,35]. Adaptive fusion techniques can also emphasize features that are less affected by stressful environments, for instance, gait features under high motion or EEG features under cognitive overload [36].

The presentation attack detection (PAD) process has been revolutionized by artificial intelligence, and deep learning models are now able to detect minute discrepancies in the spoofed signal [37]. The adversarially robust architectures, namely defense distillation and input sanitization, are being incorporated into the biometric systems to mitigate vulnerabilities to adversarial machine-learning attacks [38].

Biometric-enabled IoT platforms are increasingly becoming tactical tools, integrating identity verification in drones, exosuits, and edge devices [39]. The platforms allow continuous authentication in distributed networks, thereby ensuring secure entry into mission-critical systems in contested environments.

### 6.2. Defense-Grade Wearables and Sensor Platforms

**Figure 6:**
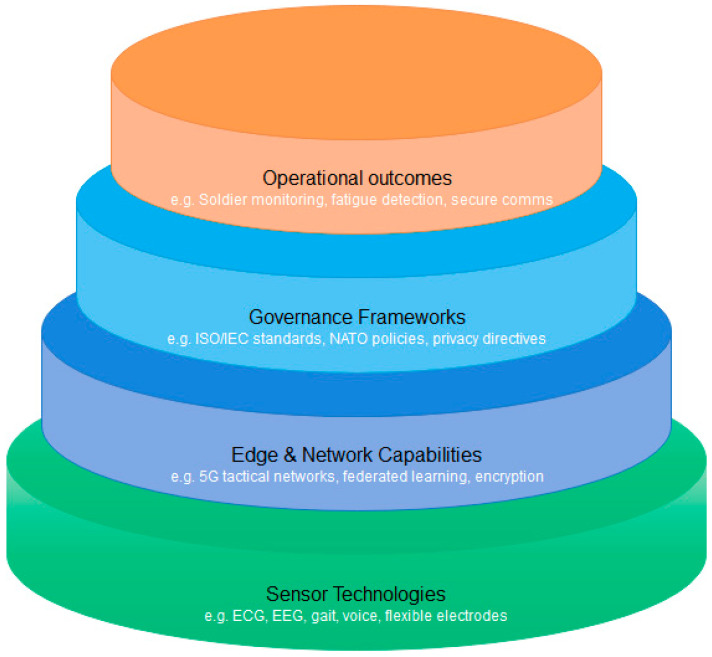
Multi-layered architecture of military-grade biometric wearables.

Tactical-grade wearables are rapidly transforming into enabling systems for biometric verification and monitoring of soldiers. Following the discussion of biometric modalities and operational stressors, this section focuses on innovation trends related to the integration of biometrics into ruggedized systems. Reflecting these innovation trends, Figure 6 depicts the multi-layered architecture supporting biometric integration in ruggedized military wearables.

The use of biosensors embedded in smart fabrics today enables the non-invasive and automatic recording of ECG, hydration, and thermal levels without relying on external, portable devices [40]. Exosuits and tactical vests are being designed with dry EEG electrodes and IMUs, allowing non-invasive and continuous observation of fatigue and cognitive functions [41].

ECG patches and PPG ring solutions offer low-power and robust monitoring capabilities, and they are designed to be resilient in high temperatures, dust, and motion [42]. Rather than being incremental, this technology is leading towards platform-integrated wearables, which will act as platforms to carry out identity verification, health, and awareness processes through their multifaceted functionality.

Edge computing infrastructure, such as tactical cloudlets and federated learning systems, enables the local processing of biometric data, thereby reducing latency and cyber risks [43]. The Science & Technology Organization of NATO has identified wearables as essential in monitoring the performance of soldiers, combining authentication with readiness [44].

In this manner, military-grade wearables are marketed as future enablers, integrating biometric interfaces into robust systems, well beyond simple authentication, and into more holistic support for soldiers.

### 6.3. Integration Pathways and Readiness Gaps

Despite technological progress, significant gaps remain in transitioning from laboratory prototypes to field-ready systems. Current biometric wearables often lack interoperability across allied forces, creating fragmentation in deployment [45]. Validation protocols are inconsistent, with few standardized stress-test datasets available for military contexts [46].

Bridging these gaps requires NATO-aligned benchmarks and governance frameworks, as outlined in the NATO Data Exploitation Framework Policy (2022), ensuring that biometric systems are tested under realistic operational conditions [47]. Integration pathways must also address legacy systems, enabling backward compatibility with existing identity management infrastructures.

### 6.4. Ethical, Legal, and Governance Frameworks

Use of biometric wearables is accompanied by very deep ethical and legal issues. Guidelines for lifecycle governance are offered by ISO/IEC 24714:2023, with transparency, proportionality, and accountability as cornerstones in managing biometric systems [48]. NATO ACT’s Personal Information and Privacy Protection Directive (2022) has outlined the responsibilities in managing personal information, biometric data, among others, to comply with the standards of privacy, security, among others, in multinational operations [49].

Risk-sensitive models of privacy recommend adaptive governance, striking a balance between operational security and the right to privacy [50]. The creation of ethics boards for biometric issues in defense institutions will enable monitoring to remain above spying [51].

### 6.5. Strategic Recommendations and Emerging Directions

To ensure safe and ethical utilization of biometric wearables in the defense sector, the following are some of the most important and urgent technology roadmaps. Firstly, it is important to develop multimodal fusion platforms, which will support dynamic weighting and switching based on various biometric modes, ultimately promoting increased robustness and accuracy under operational uncertainties [52]. Side by side, it is important to develop common protocols on presentation attack detection (PAD), which should allow equal assessments to various modes to prevent potential degradations under stress conditions [6,31].

To effectively mitigate potential adversarial attacks, it is important to develop models resistant to spoofing, which is possible by the creation of synthetic attack sets and utilization of techniques such as federated learning, which enhance robustness without violating data privacy [53]. Moreover, to ensure optimal defense against adverse utilization, it is important to establish governance to support the role of biometric ethics boards to monitor and control consent, governance, and appropriate utilization of biometric technology, ultimately promoting the role of embedded ethics and accountability during operational procedures [32,51]. Ultimately, interoperability and controls should also improve by collaborating with benchmarks and ISO-IEC-SC 37, which are universally accepted tools and platforms to encourage suitable assessments and integration of evaluation and assessment of various biometric systems [33,48].

The next generation of military biometrics will be integrated into wearables, where multimodal fusion, AI-enhanced PAD, and edge computing can provide secure, always-on authentication. But while innovation needs to be complemented by well-governed, standardized, and strategically invested efforts, the adoption of biometrics in the military wearable domain will help the military achieve resiliency and trustworthiness in terms of the management of authentication.

## 7. Conclusions

It has become apparent in conducting this analysis that there is significant scope in understanding biometric identification and tactical-grade wearable technology in its totality, which encompasses its underlying principles, functionalities, and governance models. Work in electrocardiography, electroencephalography, gait analysis, and voice identification has required a clear understanding of the underlying physiology, signal-processing demands, and the practical conditions under which these modalities can serve as reliable identifiers. In addition, the development of wearable systems—from flexible sensor designs to on-device and edge-level processing—has formed an essential part of evaluating their suitability for operational use.

The analysis further examined interoperability and governance issues for biometric wearables, considering international standards and relevant defense policies. ISO/IEC played an instrumental role in conducting methodologically grounded performance testing, coupled with NATO’s Data Exploitation Framework Policy, which emphasized not only lawful data management in international contexts, but also highlighted data privacy. These strategic imperatives have now received appropriate support from civil governance bodies such as the Centre for Information Policy Leadership, emphasizing the importance of risk-based governance for biometric wearables.

Throughout the manuscript, the discussion has underlined the inherent complexity spanning sensing technologies, network architectures, governance frameworks, and operational performance. Tactical-grade biometric wearables are not merely hardware artifacts; they function as socio-technical systems whose effectiveness depends on physiological measurement fidelity, computational integrity, and adherence to appropriate governance structures. Their integration across these dimensions determines their utility in applications such as fatigue assessment, secure communications, and force readiness, while ensuring compliance with applicable international standards and regulatory frameworks.

In conclusion, this work argues that biometric authentication in military applications will depend not only on advances in sensing technologies but also on the establishment of regulatory frameworks that ensure ethical and accountable use. When these elements are in place, biometric wearables can serve as dependable tools that support operational performance and readiness, without being undermined by ethical or societal concerns.

## Figures and Tables

**Table 1:** Comparison of sensor types in military wearables [1,3,7,8,9,10,11,12,13,14,15,16].

Sensor Type	Functionality	Defense Use Case	Field Challenges
ECG	Cardiac monitoring, biometrics	Stress detection, identity verification	Motion artifacts, electrode placement
PPG	Blood volume pulse detection	Heart rate, exertion tracking	Perfusion variability, motion sensitivity
EEG	Brain activity	Fatigue, cognitive load	Noise, helmet integration
EMG	Muscle activation	Exoskeleton control, strain prediction	Sweat, armor interference
IMU	Kinematic motion and vibration capture	Gait analysis, navigation	Drift, gear interference
Environmental	External condition sensing	Heart rate, exertion tracking	Calibration drift, sensor durability

**Table 2:** Key biometric modalities in military authentication [1,3,16,17,18,19,20,21,22,23,24].

Sensor Type	Data Source	Strength	Limitation
ECG	Heart signals	Unique, continuous	Motion artifacts
PPG	Blood pulse	Low-power, wearable	Motion noise
EEG	Brainwaves	Hard to spoof	Noise, privacy
EMG	Muscle activity	Neuromuscular specific	Sweat, electrode shift
Gait	IMU patterns	Unobtrusive	Load/terrain effects
Voice	Vocal features	Hands-free	Spoofing, noise
Fusion	Multi-signals	Robust, redundant	Integration complexity

**Table 3:** Gear interference and its impact on biometric modalities in military contexts [3,8,11,12,14,20,22,23].

Gear Type	Affected Modality	Impact on Signal Quality
Helmet/Visor	EEG, Voice	Electrode obstruction; muffled voice capture
Gloves/Exosuits	PPG, EMG, Gesture	Blocked optical sensors; reduced muscle signal capture
Load-bearing Vest	ECG, IMU	Strap displacement; altered gait dynamics
Smart Uniforms	Multimodal (ECG, PPG, IMU)	Motion artifacts; misalignment of embedded sensors
Weapon Systems/Gear	IMU, Gait	Vibration and recoil noise

**Table 4:** Security and privacy threats in military biometric authentication and corresponding countermeasures [6,30,31,32,33].

Threat Category	Examples	Impact	Countermeasures
Template Theft/Inversion	Database breach, reconstruction of ECG/EEG	Identity compromise, irreversibility	Cancelable biometrics, homomorphic encryption
Spoofing/Presentation Attacks	Replay ECG/PPG, gait imitation, voice deepfake	False acceptances, impersonation	PAD (ISO/IEC 30107), liveness detection
Adversarial ML	Perturbations, model inversion, poisoning	Misclassification, denial of service	Robust training, input sanitization
Privacy Intrusion	Fatigue/stress profiling, continuous monitoring	Autonomy loss, surveillance misuse	Ethical governance, informed consent
Standards Gaps	Fragmented military guidelines	Lack of interoperability, trust	ISO/IEC SC 37 roadmap, NATO directives

## Data Availability

No new data were created or analyzed in this study.

## Abbreviations

The following abbreviations are used in this manuscript:

AI	Artificial Intelligence
IoT	Internet of Things
ECG	Electrocardiography
EEG	Electroencephalography
EMG	Electromyography
PPG	Photoplethysmography
IMUs	Inertial Measurement Units
R&D	Research and Development
ISO/IEC	International Organization for Standardization/International Electrotechnical Commission
NATO	North Atlantic Treaty Organization
ACT	Allied Command Transformation (NATO)
CBRN	Chemical, Biological, Radiological, and Nuclear
GPS	Global Positioning System
BSNs	Body Sensor Networks
CJADC2	Combined Joint All-Domain Command and Control
IoBT	Internet of Battlefield Things
PAD	Presentation Attack Detection
